# Supplementary material for: Stability of blood lead levels in children with low-level lead absorption
Source: PLoS One. 2023 Jun 23;18(6):e0287406. doi: 10.1371/journal.pone.0287406 (PMC10289421; doi:10.1371/journal.pone.0287406)
Supplement: S2 File — (DOCX) [file pone.0287406.s002.docx]

**SPSS syntax longitudinal data analysis**

**Unconditional mean model (Level 1 Model without TIME)**

mixed BLL

/fixed intercept

/random intercept | subject(ID) covtype(un)

/print solution testcov /method ml.

**Unconditional linear growth curve model (Level 1 Model with TIME)**

mixed BLL with TIME

/fixed intercept TIME

/random TIME | subject(ID) covtype(un)

/print solution testcov /method ml.

**Adding predictors (Level 2 model with AGE and SEX)**

mixed BLL with AGE SEX

/fixed intercept AGE SEX AGE*SEX

/repeated TIME | subject(ID) covtype(un)

/print solution testcov /method ml.

**Adding Predictors (Level 2 model with AGE, SEX, and POVERTY)**

mixed BLL with AGE SEX POVERTY

/fixed intercept AGE SEX POVERTY AGE*SEX POVERTY*AGE POVERTY*SEX POVERTY*AGE*SEX

/repeated TIME | subject(ID) covtype(un)

/print solution testcov /method ml.

**Adding Predictors (Level 2 model with AGE, SEX, and OLDER_HOME)**

mixed BLL with AGE SEX OLDER_HOME

/fixed intercept AGE SEX OLDER_HOME AGE*SEX OLDER_HOME*AGE OLDER_HOME*SEX

OLDER_HOME*AGE*SEX

/repeated TIME | subject(ID) covtype(un)

/print solution testcov /method ml.

**Adding Predictors (Level 2 model with AGE, SEX, and INDUSTRY)**

mixed BLL with AGE SEX INDUSTRY

/fixed intercept AGE SEX INDUSTRY AGE*SEX INDUSTRY*AGE INDUSTRY*SEX

INDUSTRY*AGE*SEX /repeated TIME | subject(ID) covtype(un)

/print solution testcov /method ml.

**For more information contact Michelle Del Rio at midelrio@iu.edu.**
