# Supplementary material for: Stability of blood lead levels in children with low-level lead absorption
Source: PLoS One. 2023 Jun 23;18(6):e0287406. doi: 10.1371/journal.pone.0287406 (PMC10289421; doi:10.1371/journal.pone.0287406)
Supplement: Attachment — Submitted filename: ReviewPONE-D-22-34079_Mahbubur Rahman.docx [file pone.0287406.s003.docx]

Very well written, and I appreciate this important work. This is a very important work for the sectors. I like the way the findings are presented. However, a few minor comments might improve the article” Stability of blood lead levels in children with low-level lead absorption”

1. It would be great to inform sample transportation time from collection to lab (line 157). In addition, having a very brief of data collectors and their qualifications.
2. Adding a sentence on policy implications would enrich the article and may be included in the conclusion of the discussion section.
3. As the study didn’t look at the impact of interventions discussed in the manuscript, do they want to recommend any future research?
