# Supplementary material for: Stability of blood lead levels in children with low-level lead absorption
Source: PLoS One. 2023 Jun 23;18(6):e0287406. doi: 10.1371/journal.pone.0287406 (PMC10289421; doi:10.1371/journal.pone.0287406)
Supplement: Attachment — Submitted filename: DelRio_Responsetoreviewers_29May2023.docx [file pone.0287406.s004.docx]

May 29, 2023

Shailja Sharma

Acadmic Editor

PLOS ONE

Dear Shailja Sharma:

Thank you for forwarding the reviewers’ comments for our manuscript entitled “Stability of blood lead levels in children with low-level lead absorption” submitted for review to PLOS ONE. We have incorporated all suggestions by both reviewers. We believe these revisions have resulted in an improved manuscript. Below are reviewers orginal comments followed by our response to each comment.

**Reviewer 1:**

1) *It would be great to inform sample transportation time from collection to lab (line 157). In addition, having a very brief of data collectors and their qualifications.*

After formatting manuscript to PLOS ONE template, line 157 became line 140. We have incorporated the requested information in lines 140 - 145.

2) *Adding a sentence on policy implications would enrich the article and may be included in the conclusion of the discussion section.*
3) *As the study didn’t look at the impact of interventions discussed in the manuscript, do they want to recommend any future research?*

We added a short paragraph in our discussion section to include recommended future research and policy implications of our study, lines 431 - 436.

**Reviewer 2:**

1) *Shorten abstract and need proofreading for grammatical and syntax errors.*

We have made our abstract more consise, and proofread our manuscript for grammartical and syntax errors.

**We also implemented the following changes:**

- Updated formatting for Figs 1 and 2 to comply with the PLOS ONE template.
- Updated manuscript style to comply with PLOS ONE template.
- Reviewed our reference list for completeness and included two new references [41,50].
- Uploaded the study’s minimal underlying data set and syntax coding for models as supporting information files to support reproducibility of results.
- Revised name of one author, Chandima Wekumbura.

We confirm again that this work is original, has not been published elsewhere, and is not currently under consideration for publication elsewhere. The authors have no conflicts of interest to disclose.

I am the corresponding author for this manuscript; my contact information is provided below.

Thank you for your consideration,

Michelle Del Rio, M.P.H., Ph.D.

Assistant Professor

Innovation Center, Room 294

2719 E. 10th Street

Bloomington, IN 47405

(812) 856-4106

[midelrio@iu.edu](mailto:midelrio@iu.edu)
